# From Wild Vegetable to Renal Protector: The Therapeutic Potential of *Aralia elata* (Miq.) Seem

**DOI:** 10.1002/fsn3.71212

**Published:** 2025-11-17

**Authors:** Jingyi Wu, Yue Liu, Ziyun Xu, Huifeng Tan, Min Huang, Chunbo Jiang, Weiming He, Minggang Wei, Zhenfang Du, Sheng Qiang

**Affiliations:** ^1^ Department of Nephropathy Zhangjiagang Traditional Chinese Medicine (TCM) Hospital Affiliated to Nanjing University of Chinese Medicine Zhangjiagang Jiangsu China; ^2^ Translational Medical Innovation Center Zhangjiagang TCM Hospital Affiliated to Nanjing University of Chinese Medicine Zhangjiagang Jiangsu China; ^3^ Department of Nephrology Suzhou Hospital of Traditional Chinese Medicine Suzhou Jiangsu China; ^4^ Division of Nephrology Affiliated Hospital of Nanjing University of Chinese Medicine, Jiangsu Province Hospital of Chinese Medicine Nanjing Jiangsu China; ^5^ Traditional Chinese Medicine Department The First Affiliated Hospital of Soochow University Suzhou Jiangsu China

**Keywords:** *Aralia elata* (Miq.) seem, chronic kidney disease, ferroptosis, natural food, oxidative stress

## Abstract

*Aralia elata* (Miq.) Seem. (*A. elata*) is a medicinal and edible wild vegetable, which has potential applications in the pharmaceutical and health food industries. Araloside A (Ara A) is a natural triterpenoid saponin extracted from *A. elata*. Chronic kidney disease (CKD) is an important public health problem worldwide. There is still a lack of effective drugs to delay the progression of renal injury. We experimentally found that Ara A can alleviate pathological renal injury, reduce proteinuria and improve renal dysfunction in model mice. This is associated with its ability to mitigate oxidative stress and regulate ferroptosis‐related proteins. In vitro experiments, we found that Ara A can inhibit ferroptosis in podocytes and protect functional proteins. Mechanically, Ara A alleviated iron accumulation and lipid peroxidation in vivo and in vitro, and played a renal protective role by influencing the solute carrier family 7a member 11 (SLC7A11)/glutathione (GSH)/glutathione peroxidase 4 (GPX4) signaling pathway. Through cell and animal experiments, we have verified that Ara A, a component derived from *A. elata*, can delay glomerulosclerosis, with the inhibition of oxidative stress and ferroptosis as its potential mechanisms of action. These results provide evidence for the utilization of *A. elata* as a nutraceutical for the treatment of CKD.

## Graphical abstract

*Aralia elata*, a medicinal and edible wild vegetable, yields Araloside A (Ara A), a natural triterpenoid saponin. Experimental results show that Ara A, by mitigating oxidative stress and regulating ferroptosis‐related proteins via the SLC7A11/GSH/GPX4 pathway, can alleviate renal injury, reduce proteinuria, and delay glomerulosclerosis in model mice, indicating its potential as a nutraceutical for chronic kidney disease treatment.

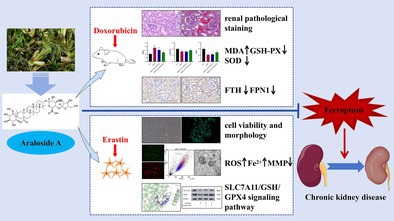

**Abbreviations**
*A. elata*
:
*Aralia elata* (Miq.) Seem
ACR: albumin/creatinine ratio
Ara A: Araloside A
CCK‐8: Cell counting Kit‐8
Ccr: creatinine clearance ratio
CKD: chronic kidney disease
DMEM: Dulbecco's modified eagle medium
DMSO: Dimethyl sulfoxide
DOX: doxorubicin
ELISA: enzyme‐linked immunosorbent assay
ESRD: end‐stage renal disease
FPN1: ferroportin1
FTH: ferritin heavy chain
GBD: Global Burden of Disease
GPx: glutathione peroxidase
GPX4: glutathione peroxidase 4
GSH: glutathione
H&E: hematoxylin and eosin
H_2_O_2_
: Hydrogen peroxide
MDA: malondialdehyde
MMP: mitochondrial membrane potential
MPC5: mouse podocyte clone 5
PAS: periodic acid‐Schiff stain
PBS: phosphate‐buffered saline
ROS: reactive oxygen species
Scr: serum creatinine
SLC7A11: solute carrier family 7A member 11
SOD: superoxide dismutase
TCM: traditional Chinese medicine
xCT: system Xc‐

## 1 Introduction

CKD is recognized as one of the global public health issues, seriously affecting people in many developed and developing regions, and imposing a huge economic burden on society (Xie et al. 2018). A systematic study from the Global Burden of Disease (GBD) revealed that there were 697.5 million CKD cases worldwide in 2017, with China (132.3 million) and India (115.1 million) accounting for a third of the total patients (GBD Chronic Kidney Disease Collaboration 2020). The early pathological features of CKD mainly include podocyte loss, glomerular hypertrophy, change of the glomerular basement membrane, and tubular damage. The final pathological manifestations of CKD are glomerular sclerosis and renal interstitial fibrosis (Webster et al. 2017). At present, the treatment of CKD mainly relies on anti‐inflammatory and anti‐renin‐angiotensin‐aldosterone system drugs, and kidney replacement therapy is the main treatment when it progresses to end‐stage renal disease (ESRD) (Webster et al. 2017). The cost of treatment is high, and the treatment cannot fundamentally delay the progression of CKD. Hence, it is of great clinical value to make clear the specific molecular mechanism of the occurrence and development of CKD and to find drugs for corresponding targets.

Numerous studies have demonstrated that the renal pathological changes in CKD patients caused by various reasons are related to oxidative stress (Duni et al. 2019; Kishi et al. 2024). The damage of podocytes is the most important early event in the progression of CKD (Zhou et al. 2019). Podocytes are known to be particularly sensitive to oxidative stress and oxidative stress caused by excessive reactive oxygen species (ROS) can lead to podocyte damage or dysfunction (Gorin and Wauquier 2015; Piwkowska 2017; Tan et al. 2015). Consequently, the production of excess ROS and the mode of cell death linked with oxidative stress have been the research hotspot.

Ferroptosis is a type of iron‐dependent cell death that is mostly brought on by abnormal iron metabolism and a significant buildup of lipid peroxides (Jiang et al. 2021). Recent studies have revealed that podocyte injury is closely related to ferroptosis and GPX4 is an important regulator of ferroptosis (Wu et al. 2022; Zhang, Swanda, et al. 2021; Zhang, Hu, et al. 2021). It can be found that signs of ferroptosis such as reduced GPX4 expression, uplifted iron content, and ROS levels can be observed in high‐sugar‐induced podocyte injury (Chen et al. 2022). Therefore, investigating the function and associated mechanism of ferroptosis in podocyte injury can serve as a foundation for the creation of specific medications.

*Aralia elata* (Miq.) Seem. (*A. elata*) is a medicinal and edible plant widely distributed in China, Japan and South Korea. *A. elata* was first known as “Congmu” in “Supplement to Medica” (Chen Tsang‐chi, in 739). Now, it is usually called “Ci long ya” in China. Due to its rich nutrition and delicious taste, *A. elata* has become a precious wild vegetable loved by people in East Asia (Xia et al. 2021). It is of great significance to develop it into a healthy food with therapeutic benefits. Triterpenoid saponins are the main bioactive components of *A. elata* (Wang et al. 2022). Ara A is a triterpenoid saponin isolated from *A. elata*, which has great potential in the pharmaceutical and healthcare industries (Yang et al. 2018). It has antioxidant, anti‐inflammatory, and other effects in modern pharmacological research (Ding et al. 2019; Tian et al. 2021). However, Ara A has received less attention in the field of renal medicine. Whether Ara A can treat CKD and its specific mechanism are still unclear. In the current study, we created a doxorubicin (DOX)‐induced mouse renal injury model in vivo and an erastin‐induced podocyte ferroptosis model in vitro, and verified that Ara A may protect podocytes and postpone the progression of CKD by inhibiting ferroptosis.

The purpose of this article is to explore the impact and underlying mechanisms of Ara A, a component derived from *A. elata*, on renal injury in CKD. The results reveal that Ara A can delay glomerulosclerosis by inhibiting oxidative stress and ferroptosis, providing significant insights into the potential applications of *A. elata* in renal protection and supporting its future use as a nutraceutical in the treatment of CKD.

## 2 Materials and Methods

### 2.1 Chemicals and Reagents

Ara A for mouse podocyte clone 5 (MPC5) cells was purchased from MedChemExpress (HY‐N2115, MCE, USA). Erastin was purchased from MedChemExpress (HY‐15763, MCE, USA). Ara A for BALB/c mice was purchased from Yuanye (B20219, Shanghai Yuanye Bio‐Technology Co. LTD., China). DOX was purchased from MedChemExpress (HY‐15142, MCE, USA). The reserve solutions of Ara A (25 mM) and Erastin (22.85 mM) were prepared with Dimethyl sulfoxide (DMSO) for in vitro experiments. The corresponding working solution was prepared by diluting the reserve solution in the medium. In vivo experiments, DMSO was also used to prepare a reserve solution of Ara A (100 mg/mL), and normal saline was utilized to create a reserve solution of DOX (5 mg/mL). Similarly, the reserve solution was diluted with regular saline to create the working solution.

Besides, antibodies used for western blot analysis, immunohistochemical staining, and immunofluorescence staining included GPX4 (ab125066, Abcam, UK), ferritin heavy chain (FTH) (ab183781, Abcam, UK), ferroportin1 (FPN1) (26601–1‐AP, Proteintech, USA), SLC7A11 (ab307601, Abcam, UK), NPHS2 (ab181143, Abcam, UK), Synaptopodin (sc‐515842, Santa Cruz, USA), β‐actin (4970S, Cell Signaling Technology, USA), Anti‐rabbit immunoglobulin G (7074S, Cell Signaling Technology, USA).

### 2.2 Animals and Experimental Treatments

Twelve male BALB/c mice weighing 25 ± 5 g were bought from JOINN Laboratory, Suzhou, China (NO: 202236274) and adaptively fed for 1 week before the experiment. The mice were randomized into four groups (three mice per group): control group, DOX group, DOX + Ara A low‐dose group (1 mg/kg), and DOX + Ara A high‐dose group (10 mg/kg). After brief isoflurane anesthesia, mice of the DOX group and DOX + Ara A group were injected with DOX (11.5 mg/kg) through the tail vein to create a DOX nephropathy model (Wu et al. 2021). In contrast, an identical volume of normal saline was injected into the mice in the control group. After 24 h, Ara A was administered intraperitoneally to the mice in the DOX + Ara A group, while mice in the control group and the DOX group received an equal volume of normal saline via intraperitoneal injection. Thereafter, the intraperitoneal injection procedure was repeated every other day for a duration of 6 weeks. The mice were euthanized after 6 weeks under general anesthesia and serum and kidney tissue were taken for further examination. The kidneys were freed from fatty adherents and were weighed to calculate the kidney/body weight ratio. Animal studies were reviewed and approved by the Animal Ethics and Welfare Committee of Zhangjiagang TCM Hospital Affiliated to Nanjing University of Chinese Medicine (Approval No: AEWC‐202201013).

### 2.3 Serum Creatinine (Scr) Measurement and Creatinine Clearance Ratio (Ccr) Calculation

The blood sample was obtained by removing the left eyeball of the mouse and then kept at room temperature for 1 h. Centrifugation (3500 rpm, 10 min, 4°C) was used to obtain serum for subsequent investigations. The Scr levels of serum were measured with a Scr kit (S03076, Rayto Institute of Biotechnology, China) and the Ccr value was calculated based on the Scr result.

### 2.4 Measurement of Albumin/Creatinine Ratio (ACR)

The urine of mice in each group was collected in a metabolic cage for 24 h beforehand death. These metabolic cages were designed with a mesh floor and an inclined collection tray, enabling the separation of feces from urine. Following collection, the urine was transferred to centrifuge tubes. Centrifugation was performed under conditions of 3000 rpm at 4°C for 10 min to sediment large particles while retaining proteins such as albumin. After centrifugation, the supernatant was collected. The urine albumin and urine creatinine of mice were measured by enzyme‐linked immunosorbent assay (ELISA) kit (20190722, Jiancheng Institute of Biotechnology, China). Albuminuria was quantified by ACR (Fu et al. 2022).

### 2.5 Histopathological Examination

The ipsilateral kidneys in each group were taken out after the cervical vertebra was severed. The kidney was then cleaned with phosphate‐buffered saline (PBS) to remove the capsule, fixed with 4% paraformaldehyde for 24 h, dehydrated progressively with 75%–100% ethanol, dipped in wax, embedded in paraffin, and finally cut into slices (4 μm). To assess renal pathological alterations, Masson, hematoxylin and eosin (H&E), and periodic acid‐Schiff stain (PAS) staining were utilized.

### 2.6 Measurement of Malondialdehyde (MDA), glutathione Peroxidase (GPx) and Superoxide Dismutase (SOD)

The aforementioned serum samples were examined by the GPx assay kit (A005, Jiancheng Institute of Biotechnology, China), SOD detection kit (S0101M, Beyotime, China), and MDA assay kit (A003‐1, Jiancheng Institute of Biotechnology, China). The MDA levels, GPx, and SOD activity were recorded at wavelengths of 530, 412, and 450 nm in a microplate reader, respectively.

### 2.7 Immunohistochemical Staining

Kidney tissue sections that had been fixed in paraffin were baked at 60°C for 45 min before being dewaxed in xylene (2 × 10 min). The dewaxed slices were first hydrated soaked in a gradient ethanol solution of 100% ethanol, 95% ethanol, 85% ethanol, and 75% ethanol, in that order. Then, the slices were cleaned with PBS thrice. Antigen repair was maintained at 120°C for 15 min in a solution of sodium citrate buffer. The specific region was circled with an immunohistochemistry pen and added with hydrogen peroxide (H_2_O_2_) which was shielded from the light for 10 min. After a second PBS wash, sections were sealed with the suitable amount of goat serum and incubated with anti‐FTH antibodies (1:400, ab183781, abcam, UK) and anti‐FPN1 antibodies (1:50, 26601‐1‐AP, proteintech, USA) at 4°C overnight. On the second day, the slices were rinsed with PBS and then incubated with biotin‐labeled goat‐resistant rabbits at room temperature for 30 min. Finally, pictures were captured with a Nikon Eclipse Ci‐L optical microscope (Nikon, Tokyo, Japan), and positive cells (brown) in a specific area were quantified.

### 2.8 Immunofluorescent Staining

After antigen repair, the paraffin‐embedded kidney tissue sections were cultured with anti‐GPX4 antibodies (1:500, ab125066, abcam, UK), anti‐SLC7A11 antibodies (1:500, ab307601, abcam, UK), and anti‐Synaptopodin antibodies (1:500, sc‐515842, Santa Cruz, USA) overnight at 4°C. The sections were subsequently treated for 30 min at 37°C with goat anti‐rabbit immunoglobulin G H&L (1:200, ab150078, abcam). Finally, the sample was stained with 4‐diaminyl‐2‐phenylindole (DAPI; C1005, Beyotime, China) and examined by a laser scanning microscope (LSM 510, Zeiss, Oberkochen, Germany).

### 2.9 Cell Culture

MPC5 cells were purchased from Beina Chuanglian Biotechnology Co. LTD. (BNCC342021, Beina, China). MPC5 cells were cultivated in Dulbecco's modified Eagle medium (DMEM) (SH30022.01, HyClone, USA) including 10% fetal bovine serum (10091148, Gibco, USA) and 100 U/mL penicillin–streptomycin–amphotericin B (C100C8, NCM Biotech, China), and placed in cell culture with 5% CO_2_ at 37°C.

### 2.10 Cell Grouping and Treatment

The MPC5 cells were cultivated in DMEM media with or without 1, 5, 10, 20, 40 μM Ara A for 24 h to see how Ara A affected the viability of MPC5 cells. Besides, to ascertain the ideal dose of erastin‐induced ferroptosis in MPC5 cells, cells were grown in DMEM media with or without 1, 3, 10, 30, or 100 μM erastin for 24 h (h). Moreover, the cell experiments were separated into four groups, containing the control group, erastin group, erastin + Ara A low‐dose group, and erastin + Ara A high‐dose group. To be specific, cells were exposed to the same volume of DMSO for 1 h and then placed in a normal DMEM medium for 24 h for the control group. For the erastin group, cells were cultured in a DMEM medium containing 3 μM erastin for 24 h after exposure to DMSO. Erastin + Ara A low‐dose group meant cells were exposed to DMEM medium containing 1 μM Ara A for 1 h and then placed in DMEM medium containing 3 μM erastin for 24 h. Correspondingly, the concentrations of Ara A and erastin were 5 μM and 3 μM for the erastin + Ara A high‐dose group, respectively.

### 2.11 Cell Counting Kit‐8 Detection

MPC5 cell suspension (5 × 10^4^ cells/mL) treated with trypsin was put into a 96‐well plate with 5000 cells per well and then transferred to the cell incubator for culture. The cells were treated by the aforementioned groups after 24 h. Then, each well received a 10 μL Cell Counting Kit‐8 (CCK‐8) solution (CK04, Dojindo, Japan) and was placed in a cell incubator for 2 h. Finally, an enzyme‐labeler (BioTek, Vermont, USA) was used to quantify absorbance at 450 nm.

### 2.12 Phalloidin Staining

MPC5 cells were inoculated on a slide and treated according to the above groups after adhesion. Thereafter, the cells were fixed in 4% paraformaldehyde (BL539A, Biosharp, China) for 30 min at room temperature. It was then incubated for 1 h at room temperature away from light with Alexa Fluor 488 phalloidin (1:20, 8878S, Cell Signaling Technology). After that, the nuclei were dyed and fixed for 10 min with a DAPI anti‐fluorescence quenching solution (P0131, Beyotime, China). Finally, the cells were photographed and observed under a fluorescent microscope.

### 2.13 Fluorescent Staining of Lipid Peroxides

The live cell assay reagent BODIPY 581/591C11 (D3861, Thermo Fisher Scientific, USA) was used to assess lipid peroxidation in cells. The differentially treated cells were rinsed twice with PBS and incubated in an incubator with 10 μM kit reagent for 30 min. The cell's fluorescence excitation wavelength shifts from 581 (red) to 500 (green) when the lipids peroxidize. The images were acquired by fluorescence microscope under uniform parameters and the green/red fluorescence ratio was calculated with ImageJ software (ImageJ 1.8, NIH, USA).

### 2.14 Determination of Intracellular Fe2+ Content

Ferroprobe FerroOrange (F374, Dojindo, Japan) was utilized to evaluate the content of ferrous ions in cells. The differentially treated cells were rinsed with cold PBS thrice, collected and suspended in a basic medium, and incubated with 1 μM ferrous probe FerroOrange (F374, Dojindo, Japan) for 15 min, and then examined by flow cytometry.

### 2.15 Transmission Electron Microscopy

Cells were immobilized using an electron microscope fixative for 2–4 h and then embedded with 1% AGAR sugar, dehydrated, and cut into slices (60–80 nm) with an ultra‐thin microtome (Leica UC 7, Leica). The slices were stained in pure ethanol with uranyl acetate for 15 min and then dyed with lead citrate for 15 min. Images were obtained using a transmission electron microscope (HT 7700, HITACHI).

### 2.16 Measurement of Mitochondrial Membrane Potential

The mitochondrial membrane potential (MMP) in MPC5 cells was detected by the JC‐1 MMP assay kit (M8650, Solarbio, China) with the manufacturer's recommendations. The differentially treated cells were rinsed with PBS before being collected and suspended in 0.5 mL complete media. After adding 0.5 mL of JC‐1 working liquid, the cells were cultured for 20 min at 37°C before being washed twice with JC‐1 buffer solution for flow cytometry analysis.

### 2.17 Western Blotting Analysis

RIPA lysis buffer (P0013C, Beyotime, China) containing a protease/phosphatase inhibitor mixture (P1045, Beyotime, China) was used to extract protein from MPC5 cells, and the concentration was measured using a BCA protein assay kit (23227, Thermo Fisher Scientific, USA). Protein samples were subjected to polyacrylamide gel electrophoresis (P0468M, Beyotime, China). Then, protein bands were transferred from the gel to a polyvinylidene fluoride membrane (IPVH00010, Millipore, USA). The membrane was then closed with a rapid sealing solution (P0252, Beyotime, China) for 15 min. After that, the membrane was treated overnight at 4°C with the primary antibodies and reacted with the secondary antibody horseradish peroxidase (7074S, Cell Signaling Technology, USA) at room temperature for 1 h on the second day. Finally, the chemiluminescent horseradish peroxidase substrate was used to visualize protein bands (WBKLS0500, Millipore, USA). The following were the primary antibodies: NPHS2 (1:2000, ab181143, abcam, UK), GPX4 (1:1000, ab125066, abcam, UK), SLC7A11 (1:1000, ab175186, abcam, UK) and β‐actin (1:1000, 4970S, Cell Signaling Technology, USA). β‐actin was used as a housekeeping gene.

### 2.18 Measurement of GSH Content

The amount of GSH in renal tissues and cells was evaluated with the GSH assay kit (ml063305V, mlbio, China). The adherent cells were rinsed once with cold PBS, digested, and collected with trypsin, and then re‐suspended in PBS containing protease inhibitor. The supernatant was then centrifuged after the cells were broken up using an ultrasonic crusher for detection. Besides, the kidney tissues were rinsed with cold PBS, weighed, ultrasonically broken following PBS homogenates containing protease inhibitors, and centrifuged for supernatant detection. The parameters of GSH were recorded at a wavelength of 450 nm in a microplate reader.

### 2.19 Statistical Analysis

GraphPad software was used to process all experimental data, which were expressed as mean ± standard deviation (M ± SD). One‐way analysis of variance was first performed among multiple groups, followed by LSD‐t test for pairwise comparisons to determine statistical significance. A *p*‐value < 0.05 was considered statistically significant.

## 3 Results

### 3.1 Ara A Improved Renal Function and Reduced Proteinuria in DOX Nephropathy Mice

**FIGURE 1:**
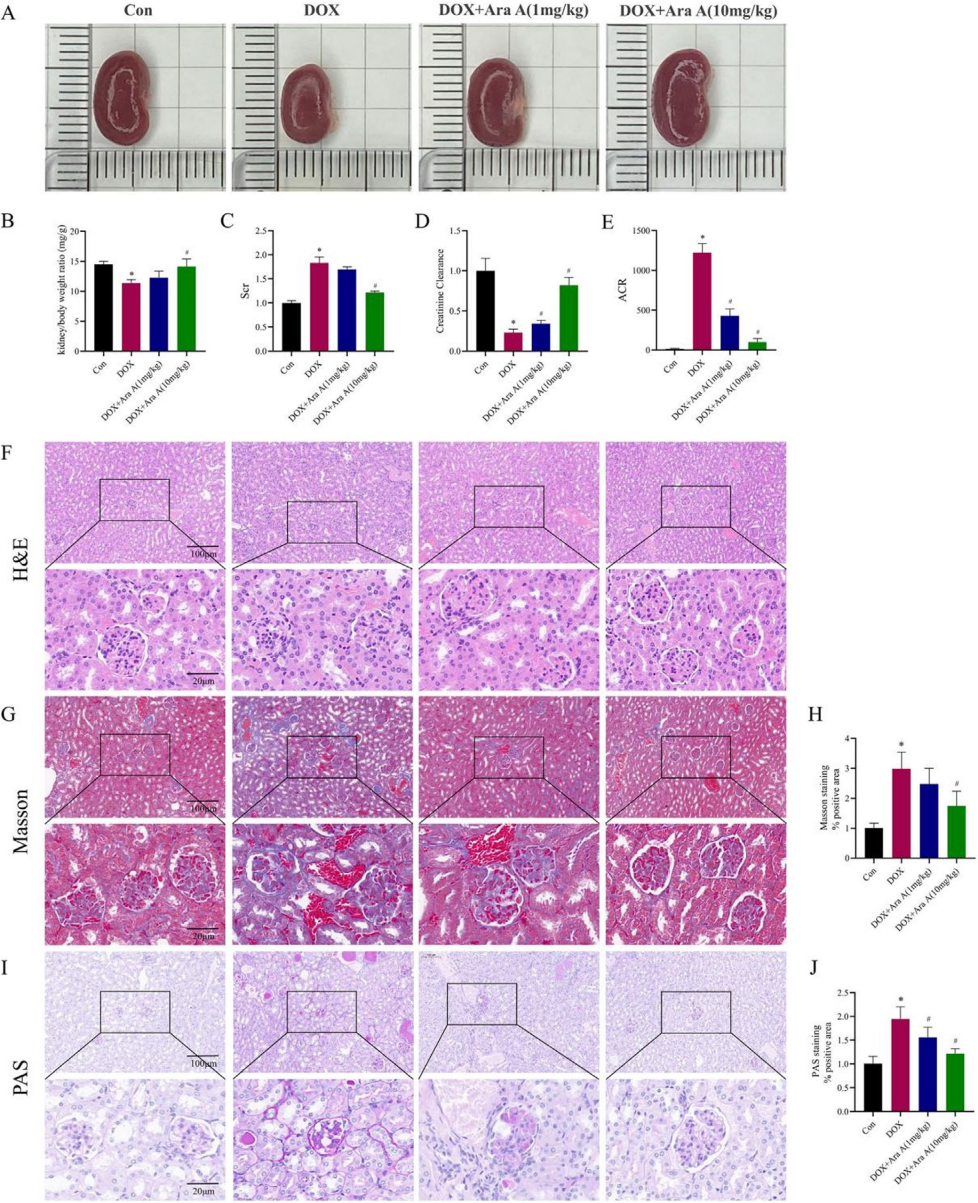
Ara A alleviated the DOX‐induced renal dysfunction and pathological changes. (A) Representative images of renal morphology of mice in each group; (B) Kidney/body weight ratio; (C) Scr level; (D) Ccr level; (E) ACR level; (F, G, I) Representative images of H&E, Masson, and PAS staining of mouse kidney sections in each group; (H, J) Semi‐quantitative analysis of Masson and PAS staining positive regions. *n* is equal to 3. *p < 0.05, compared with the control group; #*p* < 0.05, compared with the DOX group.

As a well‐known mouse model of proteinuric renal disease, DOX nephropathy exhibits severe proteinuria and impaired renal function. To determine the effects of Ara A on renal function and proteinuria, DOX was injected into the tail vein to establish a mouse model of DOX nephropathy, and then normal saline or Ara A was intraperitoneally injected into different groups of mice every other day for 6 weeks. The findings demonstrated that mice in the DOX group had higher levels of Scr and urine ACR in comparison to the control group (Figure 1C,E), whereas the Ccr value reduced (Figure 1D). We found that Ara A decreased Scr and ACR levels to a certain degree, enhanced the Ccr value, and effectively safeguarded renal function.

### 3.2 Ara A Alleviated Renal Pathological Injury in DOX Nephropathy Mice

Masson, H&E, and PAS staining were utilized to detect the pathological alterations in the kidney. Figure 1 showed that the kidneys of the DOX‐exposed mice were noticeably smaller than those of the control group mice. Further histological examination revealed glomerular distortion, glomerular adhesion, mesangial expansion, and extensive collagen deposition in the glomerular and renal interstitial areas in the kidneys of DOX‐treated mice (Figure 1F–J). In contrast, Ara A mitigated DOX‐induced pathological damage of the kidney, indicating that Ara A had a protective effect against DOX‐induced kidney damage.

### 3.3 Ara A Alleviated Oxidative Stress and Ferroptosis in DOX Nephropathy Models

**FIGURE 2:**
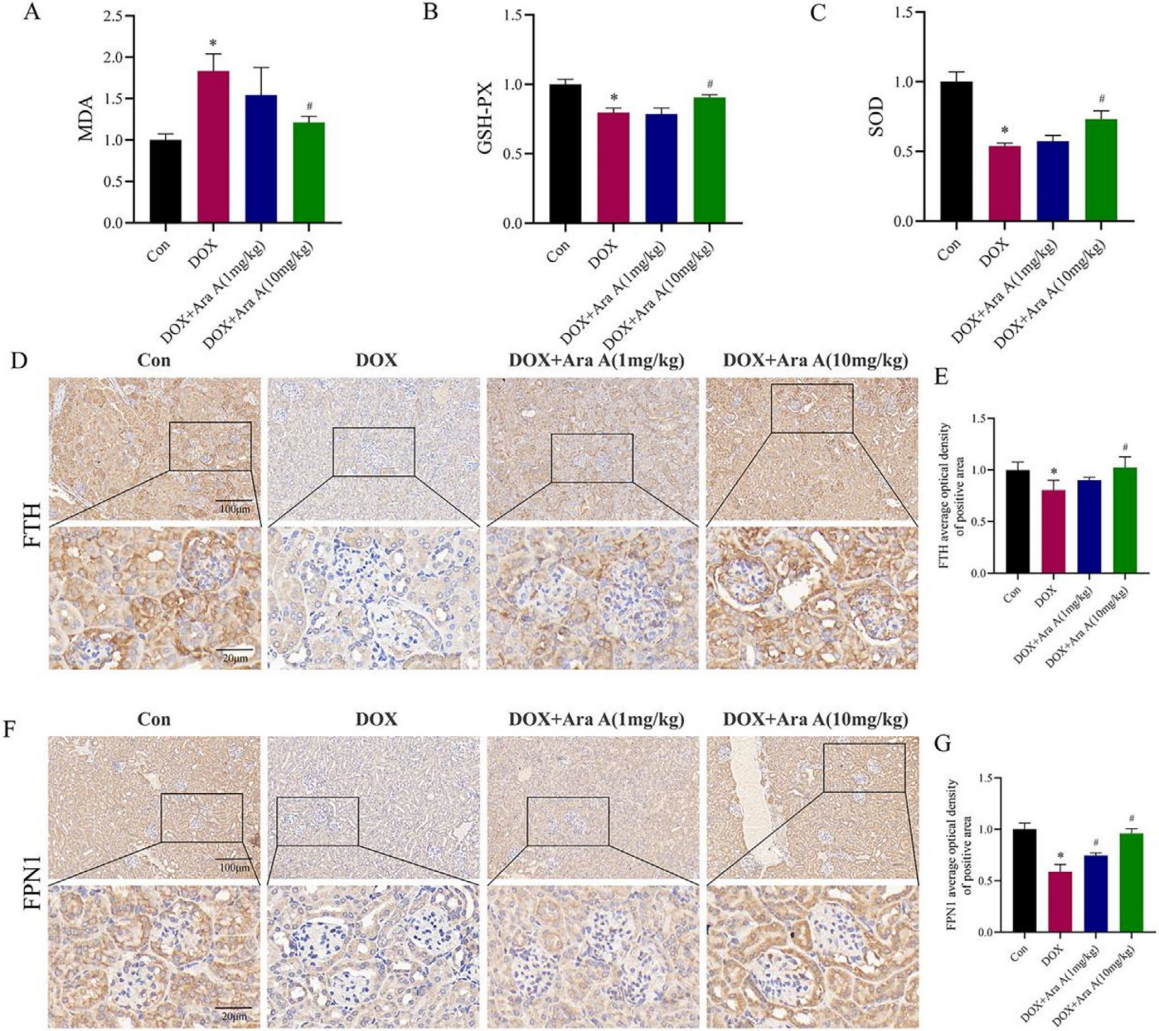
Ara A alleviated DOX‐induced oxidative stress and ferroptosis. Comparison of serum MDA level (A), GPx (B), and SOD (C) activity among groups; (D, F) representative images of FTH and FPN1 immunohistochemical staining of mouse kidney sections in each group; (E, G) semi‐quantitative analysis of FTH and FPN1 immunohistochemical positive regions. *n* is equal to 3. *p < 0.05, compared with control group; #*p* < 0.05, compared with DOX group.

The most important mechanism of DOX‐induced kidney damage is oxidative stress. To verify the influence of Ara A on DOX‐induced oxidative stress, the levels of MDA, GPx, and SOD in the serum of different groups of mice were measured. The amount of MDA was dramatically lowered by Ara A therapy, while MDA generation was observably enhanced in the DOX group in comparison to the control group (Figure 2A). Meanwhile, the antioxidant enzyme activities of GPx and SOD in the model group were significantly reduced, and the activities of GPx and SOD in the Ara A treatment group were remarkably higher compared with those in the model group (Figure 2B,C). These data indicated that Ara A inhibited oxidative stress induced by DOX. On the other hand, iron metabolism disorder is involved in the occurrence and progression of CKD. The effect of Ara A on renal iron metabolism‐related proteins in DOX‐induced kidney injury is shown (Figure 2D,F). The expressions of FTH and FPN1 were down‐regulated after DOX treatment, as demonstrated in Figure 2E,G. Ara A enhanced iron storage and output by significantly raising the expression of FTH and FPN1 in comparison to mice treated with DOX. These findings implied that Ara A may play a protective role by reducing renal iron burden.

### 3.4 The Effect of Ara A on the Activity of MPC5 Cells After Erastin Induction

**FIGURE 3:**
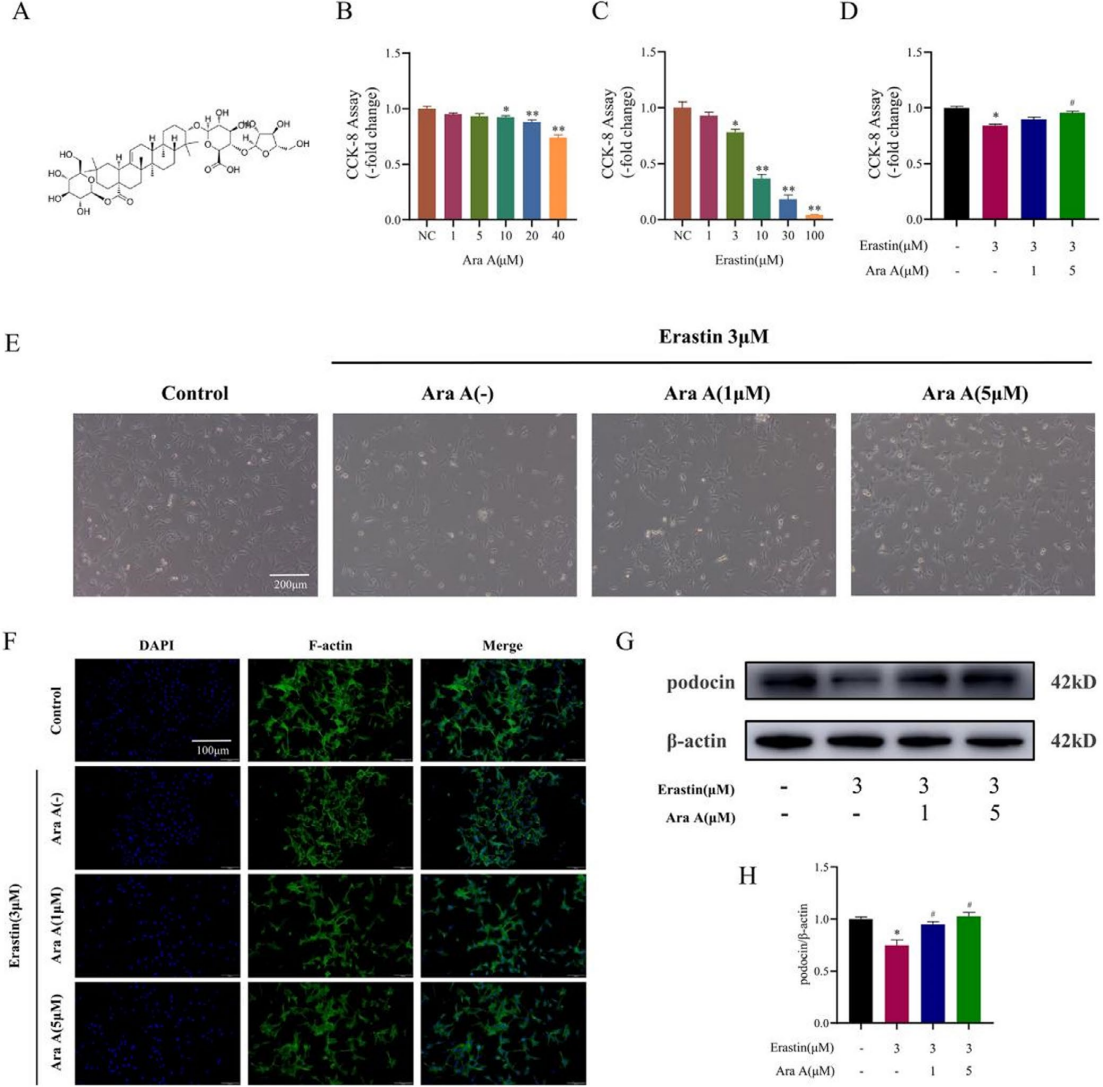
Effects of Ara A on the viability, morphology, and function of MPC5 cells after erastin induction. (A) Chemical structure of Ara A; (B, C) CCK‐8 assay was utilized to detect the cell viability of MPC5 cells treated with Ara A and erastin for 24 h; (D) MPC5 cells were pretreated with 1 or 5 μM Ara A for 1 h, and then treated with 3 μM erastin for 24 h. The activity of MPC5 cells was evaluated by CCK‐8 assay; (E) Representative optical microscope images of cell morphological changes in each group; (F) Representative images of cell phalloidin staining in each group; (G) Immunoblotting of MPC5 cell marker protein, podocin protein; (H) Semi‐quantitative analysis of podocin protein expression. *n* is equal to 3. *p < 0.05, compared with control group; #*p* < 0.05, compared with erastin group.

In vitro experiments, MPC5 cells were treated with erastin, a classical inducer of ferroptosis, to simulate iron overload in vivo. The effects of different concentrations of Ara A on MPC5 cells were first analyzed and it can be found that Ara A (1 or 5 μM) didn't affect the cell viability (Figure 3B) in comparison to the control group. The influence of various erastin concentrations on MPC5 cells was next examined (Figure 3C), and it was discovered that erastin (3 μM) was an appropriate intervention concentration, which was chosen for the following experiments. After that, MPC5 cells were cultivated in both normal medium and erastin medium with or without different concentrations of Ara A (1 or 5 μM). The results meant that the erastin group significantly inhibited cell viability in comparison to the control group, while the Ara A treatment increased cell viability to a certain extent (Figure 3D).

### 3.5 Effects of Ara A on the Morphological and Functional Proteins of MPC5 Cells After Erastin Induction

The cell morphology was observed with a microscope, and the expression of podocin, a marker protein of MPC5 cells, was detected by Western blot. After 24 h, erastin treatment significantly reduced the podocyte foot processes of the cells, and Ara A mitigated this effect (Figure 3E,F). Looking more closely at the expression of podocin, it can be found that erastin reduced the expression of podocin, and the intervention of Ara A alleviated the reduction of podocin caused by erastin (Figure 3G,H).

### 3.6 Ara A Can Reduce Erastin‐Induced Accumulation of ROS and Fe2+ in MPC5 Cells

**FIGURE 4:**
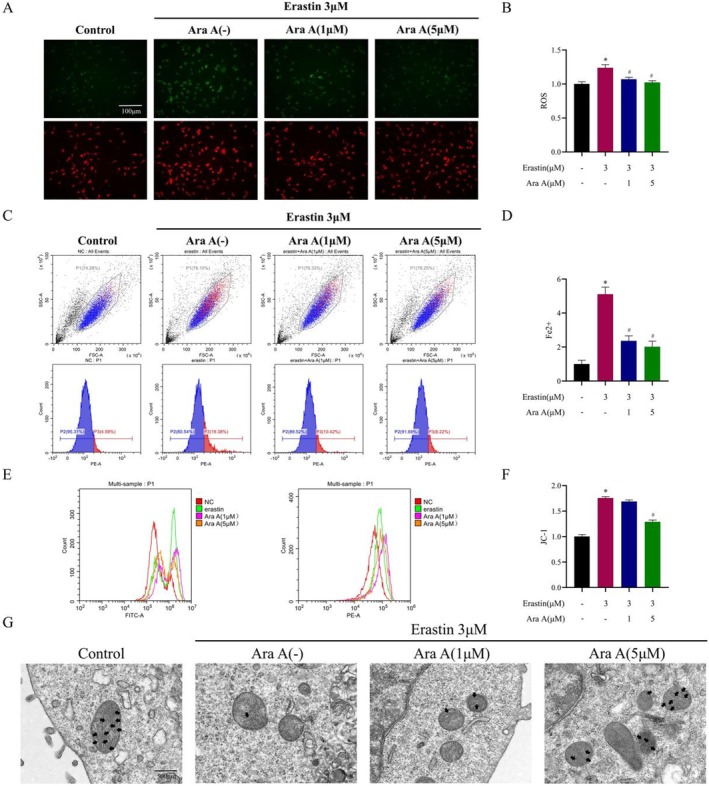
Ara A alleviates Erastin‐induced oxidative stress and ferroptosis in MPC5 cells. MPC5 cells were pretreated with 1 μM or 5 μM Ara A for 1 h and then treated with 3 μM erastin for 24 h. (A) Representative images of BODIPY 581/591C11 staining; (B) Quantitative analysis of the fluorescence ratio of green (representing lipid peroxidation)/red (conventional staining) of BODIPY 581/591 C11 staining; (C) Intracellular Fe^2+^ levels measured by flow cytometry; (D) Quantitative analysis of Fe^2+^ levels in each group; (E) Flow cytometry was utilized to evaluate MMP in each group; (F) Quantitative analysis of MMP in each group; (G) Representative transmission electron microscopy images of mitochondria in each group with black arrows representing mitochondrial cristae. *n* is equal to 3. *p < 0.05, compared with control group; #*p* < 0.05, compared with erastin group.

Ferroptosis is a type of iron‐dependent cell death caused by lipid peroxidation, so the effects of Ara A on ROS and Fe^2+^ levels in MPC5 cells were measured. Lipid peroxides rose in the erastin treatment group in comparison to the control group, and the early addition of Ara A to erastin‐induced MPC5 cells somewhat decreased ROS production (Figure 4A,B). In the physiological state, intracellular Fe^2+^ is maintained at a stable level and excess Fe^2+^ can initiate lipid peroxidation through the Fenton reaction. After testing the intracellular Fe^2+^ content, it can be observed that erastin treatment could increase the intracellular Fe^2+^ level, while Ara A could effectively lower the production of Fe^2+^ (Figure 4C,D).

### 3.7 Ara A Alleviated Erastin‐Induced Mitochondrial Morphological Changes and Membrane Potential Reduction in MPC5 Cells

Mitochondrial damage is a characteristic manifestation of ferroptosis. To study the influence of Ara A on mitochondrial damage, the mitochondrial morphology was measured by transmission electron microscopy, and changes in MMP were detected by JC‐1 staining. The results of transmission electron microscopy revealed that mitochondria shrank, membrane density increased, and the ridge shrank and vanished in the erastin group (Figure 4G), while Ara A partially alleviated the morphological changes of cell mitochondria induced by erastin. According to flow cytometry findings, erastin induced the decrease of the MMP of MPC5 cells and the increase of green/red fluorescence intensity after JC‐1 dyeing. A decline in the ratio of monomers (green fluorescence) to aggregates (red fluorescence) was observed in the MPC5 cells treated with Ara A, suggesting that Ara A prevented the MMP drop caused by erastin (Figure 4E,F).

### 3.8 The Effect of Ara A on SLC7A11/GSH/GPX4 Signaling Pathway

**FIGURE 5:**
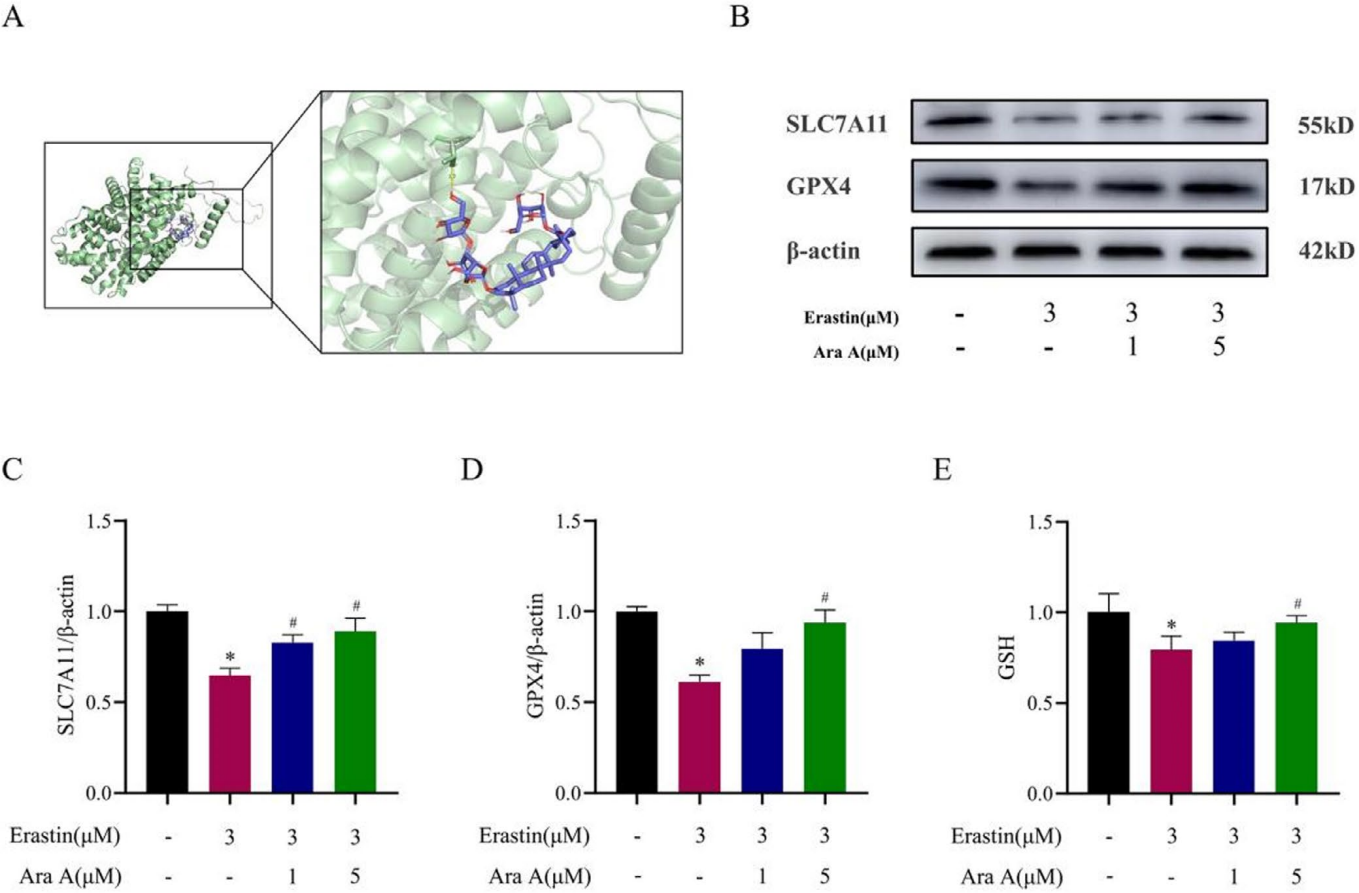
The reduction of ferroptosis by Ara A in vitro was associated with the impact of the SLC7A11/GSH/GPX4 pathway. (A) Simulation results of molecular docking between Ara A and SLC7A11; (B) western blotting results of ferroptosis‐related proteins GPX4 and SLC7A11 in each group; (C, D) semi‐quantitative analysis of GPX4 and SLC7A11 protein expression in each group; (E) GSH levels of cells in each group. *n* is equal to 3. *p < 0.05, compared with control group; #*p* < 0.05, compared with erastin group.

**FIGURE 6:**
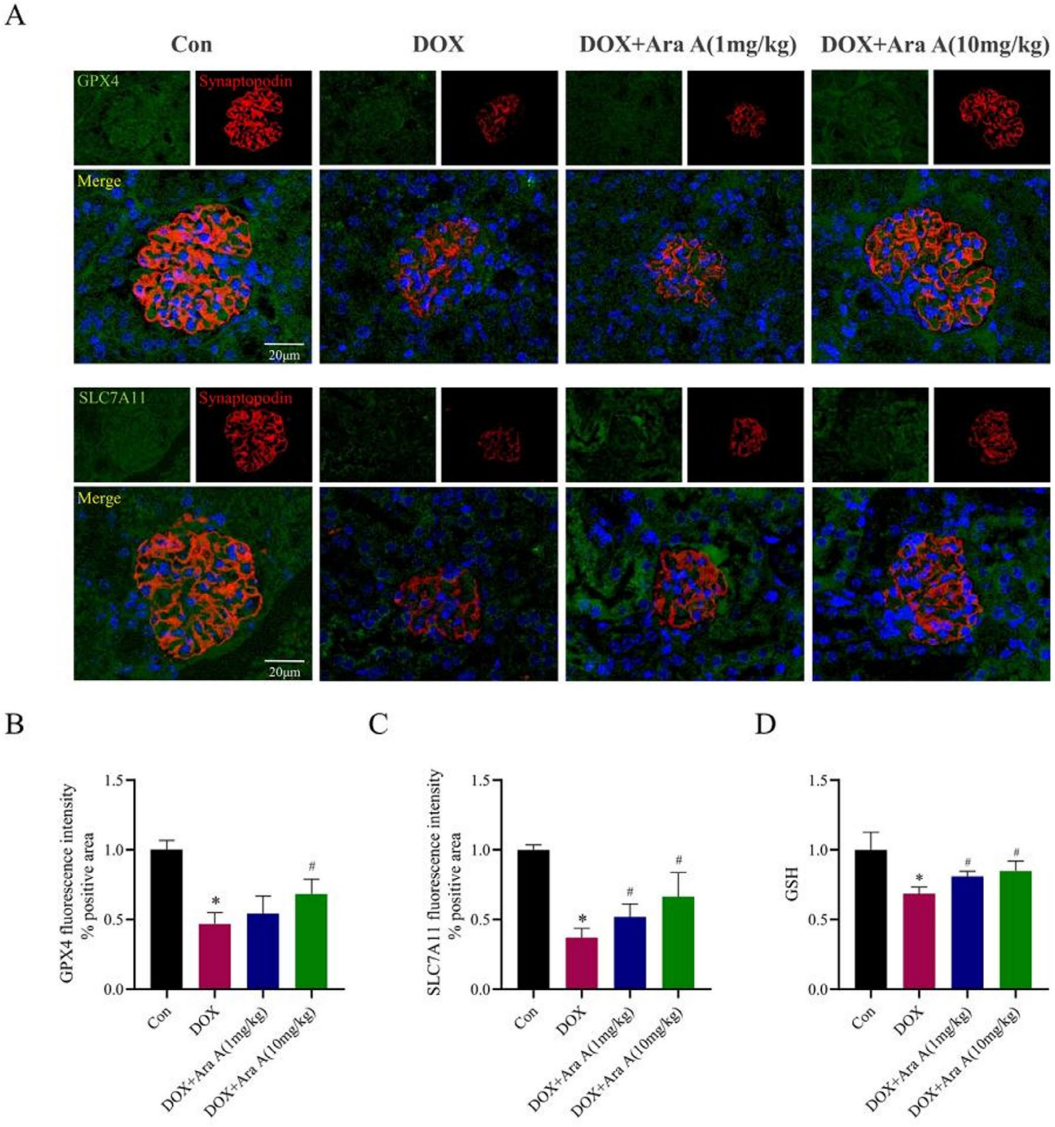
The reduction of ferroptosis by Ara A in vivo was associated with the impact of the SLC7A11/GSH/GPX4 pathway. (A) Typical images of SLC7A11, GPX4, and synaptopodin immunofluorescence staining of mouse kidney sections in each group; (B, C) semi‐quantitative analysis of the positive areas of GPX4 and SLC7A11 immunofluorescence staining in mouse kidney sections in each group; (D) GSH levels of renal tissues in each group. *n* is equal to 3. *p < 0.05, compared with control group; #*p* < 0.05, compared with DOX group.

The SLC7A11/GSH/GPX4 signaling pathway is the main pathway for erastin to induce ferroptosis in cells. To determine the influence of Ara A on the SLC7A11/GSH/GPX4 signaling pathway, it can be found that the binding of Ara A and SLC7A11 was stable by molecular docking technology and the binding energy was −6.1 kcal/mol (Figure 5A). In vitro, Western blotting was used to detect the expression of SLC7A11 and GPX4 proteins, and ELISA was employed to measure intracellular GSH levels. The results showed that, compared with the normal group, the levels of SLC7A11, GPX4, and GSH were all decreased in erastin‐induced podocytes (Figure 5B–E). Conversely, compared with the model group, the expression levels of GPX4, GSH, and SLC7A11 were increased in the Ara A group. In vivo, immunofluorescence was used to detect the expression of GPX4 and SLC7A11, and ELISA was used to measure GSH levels in renal tissues. In vivo results were consistent with in vitro findings (Figure 6A–D). These findings implied that Ara A can influence SLC7A11 and GPX4 protein expression and raise GSH levels. This may be connected to the protective effect of Ara A on erastin‐induced podocyte injury and DOX‐induced kidney injury.

## 4 Discussion

CKD is a chronic, progressive disease with abnormal kidney structure and function caused by various reasons. Its main clinical manifestations are proteinuria and renal dysfunction brought on by impaired glomerular filtration function (Inker et al. 2014; Jha et al. 2013). Glomerular podocytes are an essential component of the filtration membrane (Brinkkoetter et al. 2013; Maier et al. 2021). Podocyte damage is the primary cause of proteinuria, which is not only an important marker of kidney injury but also a substantial danger factor for the progression of CKD (Cravedi and Remuzzi 2013; Fishel Bartal et al. 2022). Unfortunately, there are currently no specific therapies that can delay the progression of CKD by targeting podocyte damage.

Podocyte injury occurs in the early stage of CKD, which is closely associated with oxidative stress (Tan et al. 2015; Zhou et al. 2019). The mechanism related to oxidative stress is the focus of research on podocyte injury, while ferroptosis may be the core mechanism to elucidate the internal relationship between oxidative stress and podocyte injury. Ferroptosis is directly tied to abnormal iron release and storage (Wu et al. 2022; Zhang, Swanda, et al. 2021). During the progression of CKD, the decrease of glomerular filtration function would cause the increase of hepcidin level, induce the internalization of membrane iron transporter protein, and affect the release of ferrous ions in podocytes (Lo et al. 2008). When excessive ferrous ions in podocytes cannot be stored in time, a large number of ROS can be generated through the Fenton reaction, resulting in podocyte damage (Yang and Stockwell 2016). The SLC7A11/GSH/GPX4 axis is a well‐known ferroptosis induction route and is closely related to intracellular antioxidant activity in podocytes (Tang et al. 2021). Concretely, as an inducer of ferroptosis, erastin can decrease the GSH level by blocking system Xc^−^ (xCT) and further affect the activity of GPX4, hence causing a rise in lipid peroxidation and ferroptosis (Ursini and Maiorino 2020). SLC7A11, one of the components of cystine‐glutamate reverse transporter xCT, is involved in GSH production and further regulates GPX4 activity through GSH, thus achieving the purpose of regulating oxidative stress (Jin et al. 2022). Besides, GPX4 is an important regulator of oxidative stress (Nemeth et al. 2004). Studies have shown that up‐regulation of GPX4 expression can protect the kidney by significantly lowering ferroptosis and oxidative stress (Chen et al. 2022; Stockwell 2022). Therefore, lipid peroxidation and ferroptosis are crucial in podocyte injury, and the search for drugs that protect podocytes by regulating oxidative stress‐related ferroptosis may provide a new approach to CKD treatment.

*A. elata* is a medicinal and edible plant, which is usually called “Ci long ya” in China. It has rich nutrition and a delicious taste. *A. elata* has become a precious wild vegetable widely loved by people in East Asia (Xia et al. 2021). The potential value of natural foods in delaying the progression of diseases is an interesting topic worth exploring. In this study, we explored the influence of Ara A, a component derived from *A. elata*, on kidney injury in CKD and confirmed that it can protect podocytes by reducing oxidative stress and ferroptosis. Figure 1 depicts our demonstration that Ara A can reduce the kidney damage caused by DOX in mice. Mice in the DOX group had smaller kidneys in volume, higher Scr and ACR levels, and lower Ccr values compared to the control group. The results of renal section staining showed that mice in the DOX group exhibited glomerular adhesion, mesangial expansion, and collagen deposition in the glomerular and renal interstitial areas, whereas Ara A could alleviate these changes to a certain extent. Subsequently, we discovered that the renal protective effect of Ara A may be linked with the reduction of oxidative stress and ferroptosis. GPx and SOD are key enzymes to prevent oxidative stress and maintain redox balance, while MDA, the product of lipid peroxidation, can cause oxidative damage (Blokhina et al. 2003; Fan et al. 2021; Khan et al. 2020). Our findings demonstrated that Ara A raised the levels of GPx and SOD, reduced the levels of MDA, and enhanced the antioxidant capacity of the mice after DOX induction. At the same time, we noticed that Ara A also has an impact on the important segments of ferroptosis. FTH can store excess iron in the cell and FPN1 is involved in the cellular excretion of iron (Rui et al. 2021; Zheng et al. 2023). So FTH and FPN1 play a vital role in iron metabolism. In vivo experiments, data proved that the expression of FTH and FPN1 in the kidney of mice decreased after DOX induction, while Ara A could alleviate these alterations to a certain extent. Therefore, it is concluded that Ara A may play a role in kidney protection by alleviating glomerular sclerosis and inhibiting ferroptosis.

Glomerular sclerosis and proteinuria are the main pathological changes and clinical manifestations of CKD. In this process, podocyte injury plays a major role (Shankland 2006). As a result, we believe that the effect of Ara A on reducing glomerular sclerosis and proteinuria may be related to protecting podocytes by inhibiting ferroptosis. In this study, podocytes treated with the classic ferroptosis inducer erastin were employed as an in vitro experimental model. It can be demonstrated from Figure 3 that Ara A can save the erastin‐induced decline in podocin activity and foot process, increase the expression of functional protein, and play a protective role in podocytes. The protective effect of Ara A may be linked to the inhibition of ferroptosis. Ferroptosis occurred in podocytes, which was manifested by decreased antioxidant capacity, increased intracellular ROS levels, Fe^2+^ accumulation, and mitochondrial damage after erastin treatment. However, the pretreatment of Ara A can effectively reduce erastin‐induced lipid peroxides accumulation, iron overload, and changes in mitochondrial morphology and membrane potential of podocytes, thus inhibiting ferroptosis.

The SLC7A11/GSH/GPX4 signaling pathway is the main pathway for erastin to induce ferroptosis (Yan et al. 2022). To explore the specific mechanism of inhibiting ferroptosis of Ara A, we found that the binding of Ara A and SLC7A11 was stable and the corresponding binding energy was −6.1 kcal/mol by molecular docking technology. Therefore, we speculated that Ara A might play a role in inhibiting ferroptosis by influencing the SLC7A11/GSH/GPX4 signaling pathway. The SLC7A11/GSH/GPX4 signaling pathway is one of the classical pathways for ferroptosis, which is mainly associated with reduced synthesis of GSH, an important intracellular antioxidant (Zhang et al. 2022). SLC7A11 is one of the component subunits of cystine and glutamate reverse transporter xCT, which is involved in promoting cystine uptake and thereby affecting the synthesis of GSH (Parker et al. 2021). GPX4 activity is positively regulated by GSH and the conversion of GSH to oxidized GSH depends on GPX4, thus inhibiting GPX4 can result in the buildup of lipid peroxides (Li et al. 2020; Yang et al. 2014). Downregulating the expression of GPX4 increases cellular sensitivity to ferroptosis (Xu et al. 2021). Our study results showed a drop in GSH levels and a decrease in cellular and tissue antioxidant capacity in the model group (in vivo and in vitro experiments). We also observed a decrease in the expression of SLC7A11 and GPX4 in the model group, while the addition of Ara A effectively increased their levels in podocytes and renal tissues. Therefore, we believe that Ara A may regulate the key molecules (GSH, GPX4) of the antioxidant system through the SLC7A11/GSH/GPX4 axis to exert antioxidant capacity and reduce ferroptosis. This regulatory property of Ara A is likely attributable to its structural basis.

Ara A is a pentacyclic triterpenoid saponin whose structural features comprise a pentacyclic triterpene skeleton and glycosyl substituents. In nature, pentacyclic triterpenoid saponins are important natural bioactive substances that are widely distributed in plants. A variety of pentacyclic triterpenoid saponins have been confirmed to exhibit antioxidant effects. Anemoside B4 can alleviate inflammatory responses by inhibiting the generation of ROS (Wu et al. 2025; Ni et al. 2025). Hederagenin can reduce ROS accumulation and cell apoptosis (Shen et al. 2023). Tea seed saponins can upregulate the levels of SOD and GSH to ameliorate oxidative stress (Cao et al. 2022). Our research has found that Ara A also exhibits antioxidant effects, which are specifically manifested by a reduction in intracellular ROS accumulation, a decrease in MDA levels, and an increase in SOD and GSH levels. Meanwhile, Ara A is also involved in regulating ferroptosis‐related proteins. Therefore, the antioxidant effects of Ara A and its regulation of the ferroptosis signaling pathway may be associated with its unique structure. Future research could focus on further investigating how the specific structure of Ara A inhibits key aspects of oxidative stress and ferroptosis, thereby elucidating the underlying mechanisms of its renal protective effects.

CKD has long suffered from a scarcity of effective clinical drugs with minimal adverse effects. Current cornerstone pharmacotherapies primarily encompass angiotensin‐converting enzyme inhibitors/angiotensin II receptor blockers, sodium‐glucose cotransporter 2 inhibitors, and aldosterone antagonists (Navaneethan et al. 2025; Zhu et al. 2023). Angiotensin‐converting enzyme inhibitors/angiotensin II receptor blockers can reduce intraglomerular pressure and decrease proteinuria by preferentially dilating the efferent arterioles through inhibition of the renin‐angiotensin system. However, they carry risks of inducing hyperkalemia and acute kidney injury. Sodium‐glucose cotransporter 2 inhibitors lower glomerular hyperfiltration via osmotic diuresis but may increase the risk of urinary tract infections (Yang et al. 2024). Aldosterone antagonists inhibit the release of inflammatory factors and renal fibrosis by blocking mineralocorticoid receptors, yet they also pose a risk of hyperkalemia and an increased likelihood of acute kidney injury when used in combination with angiotensin‐converting enzyme inhibitors/angiotensin II receptor blockers (Chung et al. 2020). Compared with conventional therapeutic agents, Ara A exhibits a distinct mechanism of action. Our research revealed that it can directly target podocytes, which are key components of glomerular cells, to exert renal protective effects. Specifically, Ara A mitigates podocyte injury by suppressing oxidative stress and ferroptosis, thereby effectively delaying disease progression. Ara A was extracted from the edible wild vegetable *A. elata*. Nevertheless, natural plants have a relatively long growth cycle, and there are limitations on the daily intake amount by the human body. How to rely on synthetic biology to achieve large‐scale and standardized heterologous synthesis of the natural compound Ara A is an important issue that needs to be addressed in the next step. Overall, our findings suggest that Ara A has the development potential as a novel renal‐protective pharmaceutical agent or functional dietary supplement, offering promising prospects for providing a new natural compound alternative therapy for patients with CKD.

## 5 Conclusion

**FIGURE 7:**
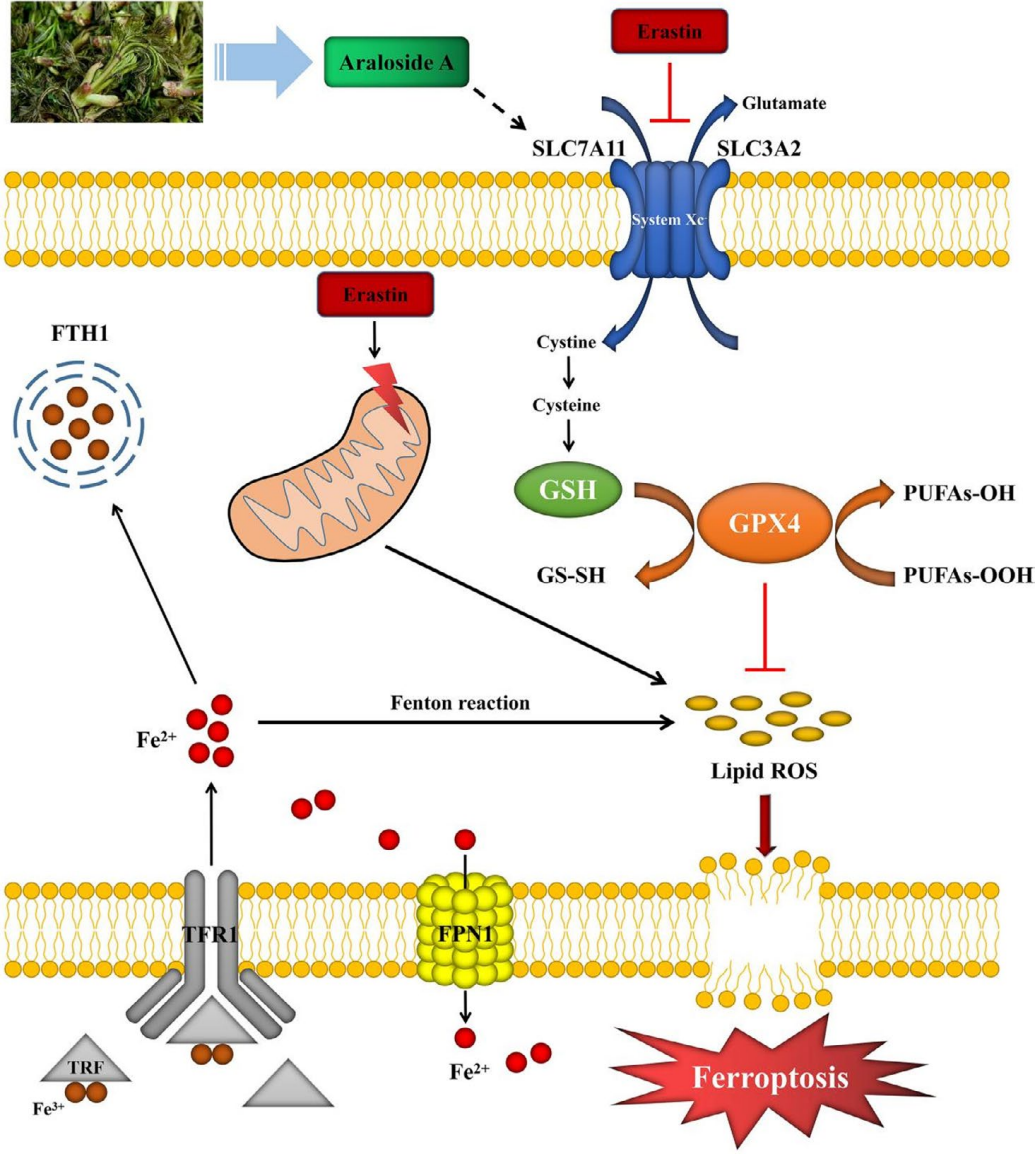
The specific mechanism of Ara A inhibiting podocyte ferroptosis.

This study found that the natural compound Ara A, derived from the wild vegetable *A. elata*, effectively protects podocyte function and kidney structure, alleviating glomerulosclerosis and proteinuria. Its protective effect is primarily achieved by inhibiting oxidative stress and ferroptosis. The experimental results demonstrated that Ara A could increase levels of key antioxidant enzymes and GSH, reduce levels of lipid peroxidation products and ROS, improve the expression of iron metabolism‐related proteins, and mitigate mitochondrial damage. Further molecular docking results and experimental data indicated that the renoprotective effect of Ara A involves the regulation of the SLC7A11/GSH/GPX4 signaling axis (Figure 7). Currently, there is a lack of specific therapeutic drugs targeting podocyte injury in clinical practice. Our study provides experimental evidence supporting the application of functional foods in CKD management, particularly for podocyte protection, and facilitates the development of food‐derived renal protective agents.

## Author Contributions

**Jingyi Wu:** conceptualization (lead), data curation (lead), investigation (lead), methodology (lead), validation (lead), writing – original draft (lead). **Yue Liu:** conceptualization (lead), data curation (lead), investigation (lead), methodology (lead), validation (lead). **Ziyun Xu:** data curation (lead), investigation (lead), visualization (lead). **Huifeng Tan:** data curation (equal), formal analysis (equal), visualization (equal). **Min Huang:** data curation (equal), formal analysis (equal), visualization (equal). **Chunbo Jiang:** data curation (equal), methodology (equal). **Weiming He:** formal analysis (equal), methodology (equal). **Minggang Wei:** resources (lead), supervision (lead), writing – review and editing (lead). **Zhenfang Du:** project administration (lead), resources (lead), supervision (lead). **Sheng Qiang:** funding acquisition (lead), project administration (lead), supervision (lead), writing – review and editing (lead).

## Ethics Statement

Animal studies were reviewed and approved by the Animal Ethics and Welfare Committee (AEWC) of Zhangjiagang TCM Hospital Affiliated to Nanjing University of Chinese Medicine (Approval NO: AEWC‐202201013).

## Conflicts of Interest

The authors declare no conflicts of interest.

## Data Availability

The data that support the findings of this study are available from the corresponding author upon reasonable request.
